# Brain Injury Impairs Working Memory and Prefrontal Circuit Function

**DOI:** 10.3389/fneur.2015.00240

**Published:** 2015-11-13

**Authors:** Colin J. Smith, Guoxiang Xiong, Jaclynn A. Elkind, Brendan Putnam, Akiva S. Cohen

**Affiliations:** ^1^Research Institute of Children’s Hospital of Philadelphia, Philadelphia, PA, USA; ^2^Neuroscience Graduate Group, University of Pennsylvania School of Medicine, Philadelphia, PA, USA; ^3^Department of Anesthesiology and Critical Care Medicine, Perelman School of Medicine, University of Pennsylvania, Philadelphia, PA, USA

**Keywords:** traumatic brain injury, lateral fluid percussion injury, synaptic transmission, intrinsic excitability, medial prefrontal cortex, working memory

## Abstract

More than 2.5 million Americans suffer a traumatic brain injury (TBI) each year. Even mild to moderate TBI causes long-lasting neurological effects. Despite its prevalence, no therapy currently exists to treat the underlying cause of cognitive impairment suffered by TBI patients. Following lateral fluid percussion injury (LFPI), the most widely used experimental model of TBI, we investigated alterations in working memory and excitatory/inhibitory synaptic balance in the prefrontal cortex. LFPI impaired working memory as assessed with a T-maze behavioral task. Field excitatory postsynaptic potentials recorded in the prefrontal cortex were reduced in slices derived from brain-injured mice. Spontaneous and miniature excitatory postsynaptic currents onto layer 2/3 neurons were more frequent in slices derived from LFPI mice, while inhibitory currents onto layer 2/3 neurons were smaller after LFPI. Additionally, an increase in action potential threshold and concomitant decrease in firing rate was observed in layer 2/3 neurons in slices from injured animals. Conversely, no differences in excitatory or inhibitory synaptic transmission onto layer 5 neurons were observed; however, layer 5 neurons demonstrated a decrease in input resistance and action potential duration after LFPI. These results demonstrate synaptic and intrinsic alterations in prefrontal circuitry that may underlie working memory impairment caused by TBI.

## Introduction

Traumatic brain injury (TBI) causes 2.5 million emergency department visits, hospitalizations, and deaths in the U.S. each year and affects millions more that do not seek medical care (1). Even mild to moderate TBI, or concussion, can cause long-lasting cognitive effects, including memory impairment (2). TBI primarily causes damage to the frontal and temporal lobes of the cerebral cortex resulting in persistent cognitive impairment (3). Functional imaging approaches have described changes to frontal lobe function following TBI (4, 5). A common complaint among TBI sufferers is a loss of working memory, a fundamental ability for maintaining quality of life after TBI (6–9). Despite the prevalence of TBI, post-injury care is palliative and no therapy exists to treat the underlying causes of the cognitive impairments suffered by TBI patients.

Lateral fluid percussion injury (LFPI) is a well characterized and routinely employed rodent model of brain injury that reproduces key features of human TBI, including neuronal cell loss, gliosis, ionic perturbation, and memory deficits (10–13). Considerable work in rodent models of TBI has focused on post-injury changes in spatial and working memory performance (13–16). A single mild to moderate LFPI has been shown to induce long-lasting memory impairments, despite a relative lack of neuronal loss, suggesting a circuit mechanism for the observed cognitive deficits (17). Furthermore, TBI-induced memory impairment has been linked to regionally specific shifts in network excitability in the hippocampus (18), and the hippocampus has been a target of therapeutic interventions designed to reinstate the balance between excitatory and inhibitory synaptic transmission (E/I balance) and restore cognition (19).

In contrast to the considerable experimental evidence on TBI-induced hippocampal pathology, there is a dearth of information regarding putative physiological changes in the prefrontal cortex after TBI. While human studies of TBI patients have shown changes, including decreased prefrontal activity (20) as well as diminished functional connectivity between hippocampus and frontal areas (21), previous research in rodent models of TBI focused on the prefrontal cortex have centered on morphological (15) or metabolic changes (22). Moreover, investigations into working memory after TBI have examined long-term impairments (>7days) and, therefore, have trained and tested experimental subjects weeks after injury (14–16). The current study is, to our knowledge, the first report to assess working memory impairment using a non-match to sample behavioral paradigm in the days immediately following mild to moderate LFPI.

Interaction between the hippocampus and the prefrontal cortex is critical for spatial working memory function in both humans and rodents (23–25). Specifically, non-match to sample behavior in rodents using the T-maze has been established as a task to evaluate working memory behavior that critically depends on contributions of the prelimbic cortex (26–28). Additionally, differences in neuronal firing patterns have been recorded in the medial prefrontal cortex (mPFC) in both primates and rodents when performing non-match to sample working memory tasks (29, 30). Altering E/I balance or specifically manipulating miniature excitatory synaptic activity (and thus E/I balance) in prefrontal cortex causes observable changes in behavior (31, 32). Layer 2/3 and layer 5 both have critical roles in the function of the prefrontal circuit as specific physiological changes in either layer cause alterations in behavior (31, 33, 34). Furthermore, it is possible that the cells in layer 2/3 and layer 5 may have differential responses to injury as is the case with layer-specific and cell-type-specific changes previously documented in the hippocampus after LFPI (18, 35–37).

In the current study, we investigated the physiological correlates of working memory dysfunction after mild to moderate LFPI. We tested for working memory deficits using a non-match to sample behavioral paradigm that critically depends on prefrontal cortex (38). Furthermore, using a physiological approach, we interrogated the underlying circuitry. That is, we investigated changes in E/I balance, synaptic transmission, and intrinsic excitability in both layer 2/3 and layer 5 of prefrontal cortex. Our results include a layer-specific series of alterations in both synaptic transmission and intrinsic excitability that may contribute to the working memory dysfunction observed over the first 7 days post-LFPI.

## Materials and Methods

### Ethical Approval

All experiments were carried out under protocols approved by the Institutional Animal Care and Use Committee of Children’s Hospital of Philadelphia and the guidelines established by the NIH *Guide for the Care and Use of Laboratory Animals*. Experiments were performed on 8- to 12-week-old male C57/BL6 mice (Jackson Laboratory, Bar Harbor, ME, USA. Stock number 000664). Experiments were designed to minimize the number of animals required and those used were cared for, handled, and medicated as appropriate to minimize their suffering. Separate cohorts of animals were used for behavioral and *ex vivo* electrophysiological experiments due to the significantly larger number of animals needed for electrophysiological experiments. A total of 120 mice were used in the experiments presented herein.

### Lateral Fluid Percussion Injury (LFPI)

After anesthetizing the animal with a mix of ketamine (2.6 mg/kg) and xylazine (0.16 mg/kg) via intraperitoneal injection, the animal was placed in a stereotaxic frame (Stoetling, Wood Dale, IL, USA), the scalp was opened, and the fascia scraped from the skull. An ultra-thin Teflon disk, with the outer diameter equal to the inner diameter of a trephine, was glued to the skull midway between Bregma and Lambda, between the sagittal suture and the lateral ridge on the right side of the skull. Using a trephine, a 3-mm diameter craniectomy was performed over the right parietal area. Following craniectomy, a Luer-lock needle hub (3 mm inner diameter) was secured above the skull opening. Finally, the animal was sutured and placed on a heating pad until mobile, at which point it was returned to its home cage. The next day, the animal was placed under isoflurane anesthesia until it reached a surgical plane of anesthesia (one respiration per 2 s). At this point, the animal was removed from isoflurane, the hub was filled with saline and connected to the fluid percussion injury device via high-pressure tubing. The animal was placed on its left side on a heating pad. Once a normal breathing pattern resumed, but before sensitivity to stimulation, the injury was induced by a brief (20 ms) pulse of saline onto the intact dura. The peak pressure was monitored with an oscilloscope and for all injuries ranged from 1.4 to 2.0 atm. Immediately after injury the hub was removed from the skull and the animal was placed in a supine position. After righting, the animal was placed under isoflurane to suture the scalp, then placed on a heating pad until mobile, at which point it was returned to its home cage. Sham animals received all of the previously described steps except the fluid pulse.

This type of LFPI is designed to produce a mild to moderate brain injury modeling a non-penetrating concussive injury, as the dura mater is not breached during the procedure. Animal righting time is used as an acute assessment of injury severity and animals with an excessive righting time were excluded from further study (39). The severity of LFPI used here results in hippocampal-dependent cognitive impairment as demonstrated by previous studies, employing contextual fear conditioning (18, 19, 40) as well as a significant reduction in neurons in all subregions of the ipsilateral hippocampus as measured using unbiased stereology (18).

### T-maze Behavioral Paradigm

**Figure 1:**
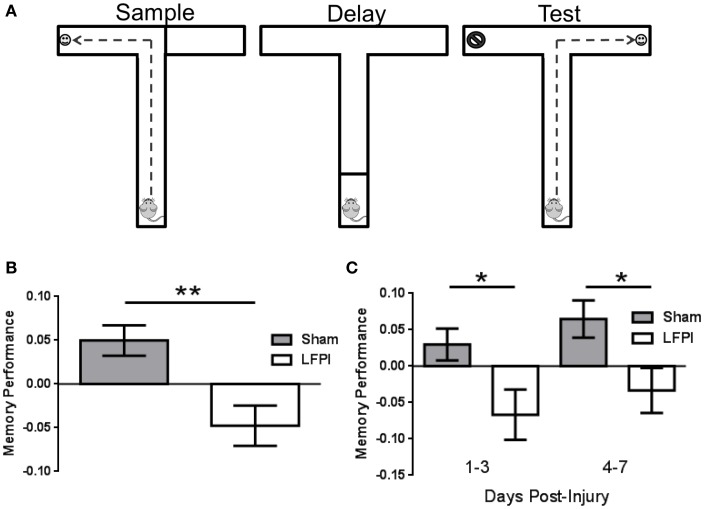
**Persistent working memory impairment immediately following LFPI**. **(A)** Schematic of the T-maze and the three phases of a trial. The sample phase exposes the animal to one arm to receive a food reward, then after the delay phase, the animal is exposed to both arms in the choice phase and must select the opposite arm in order to receive a food reward. **(B)** Memory Performance, as assessed by the delayed non-match to sample task, is impaired over the first 7 days after LFPI. (** indicates *P* < 0.001) **(C)** Memory Performance is impaired in both the first 3 days after LFPI as well as days four through seven after injury (* indicates *P* < 0.05).

In order to motivate animals for food reward, animals used in behavioral experiments were placed on a restricted diet and maintained at 90% of their free-feeding body weight for the duration of the experiment. While in the T-maze, mice were rewarded with Reese’s Peanut Butter Chips (The Hershey Company, Hershey, PA, USA). Mice were placed on the restricted diet for 3 days before beginning training, at which time they were allowed to acclimate to the T-maze for 10 min/day during a free exploration period with all parts of the maze available and no reward present. Subsequently, mice were trained on a non-match to sample behavioral task using a standard T-maze (Dimensions: all arms 7 cm wide × 12.5 cm height. Lengths: start box 17 cm, center arm 31 cm, reward arms 37.5 cm. Med Associates, St. Albans, VT, USA). Each trial consisted of a sample phase, a delay phase, and a choice phase (for schematic see Figure 1A). The animal began each trial in the start box, at the bottom of the “T.” During the sample phase either the left or right arm of the T-maze was blocked, and the animal was rewarded for exiting the start box and proceeding to the end of the maze. The animal was returned to the start box for the delay phase, which in this study was a nominal 0 s, to provide a fairly easy working memory task as in (30). The door separating the start box and the middle arm of the T-maze was lifted beginning the choice phase, during which both goal arms were available. The animal was required to choose the opposite arm to which it was exposed in the sample phase in order to obtain a reward, which it was allowed to eat for ~5 s. If it chose incorrectly, it was removed from the maze without receiving a reward. The task requires the animal to retain a memory trace of the sample phase until the point at which it selects an arm during the choice phase. An animal was determined to have chosen an arm once the entire body of the animal including tail entered the arm. Training lasted 7 days immediately preceding LFPI or sham operation after which testing took place for 7 days (10 trials/day). The 10 trials/day consisted of five left-sample and five right-sample trials, presented in pseudo-randomized order. Throughout the duration of the testing phase, the experimenter was blinded to animal condition (i.e., whether the animal had received an LFPI or sham procedure). Animals that averaged <60% correct trials over the last 3 days of training were excluded from analysis as they had not met the criterion for learning the non-match to sample rule. Sixteen mice were used for the behavioral data presented here (*n* = 9 sham-operated animals, *n* = 7 LFPI animals).

### Electrophysiology

All recordings were made 6–8 days after LFPI or sham operation. Mice were anesthetized with isoflurane, and the brains were quickly and carefully removed, then placed into ice-cold oxygenated (95% O_2_/5% CO_2_) sucrose ACSF containing (in millimolar): sucrose 202, KCl 3, NaH_2_PO_4_ 1.25, NaHCO_3_ 26, glucose 10, MgCl_2_ 1, and CaCl_2_ 2. Slices 350 μM thick were cut on a VT1200S vibratome (Leica Microsystems Inc., Buffalo Grove, IL, USA) and transferred to 33–37°C normal ACSF containing (in millimolar): NaCl 130, KCl 3, NaH_2_PO_4_ 1.25, NaHCO_3_ 26, glucose 10, MgCl_2_ 1, CaCl_2_ 2, for at least 1 h.

#### Brain Slice Selection Criteria

All experiments were performed in the prelimbic cortex, a subfield of rodent mPFC, ipsilateral to the site of brain injury or sham operation. All slices used were from Bregma +1.98 to +1.54 as determined by the presence and shape of the forceps minor of the corpus callosum (41). Under the microscope, cell layers of prelimbic cortex were easily identifiable. Layer 1 is notable for its lack of cells, while, in contrast, layer 2/3 contains a dense band of cell bodies. Moving deeper from layer 2/3 there is no layer 4 in this cortical region, while layer 5 is typified by a lower density of larger cell bodies.

#### Extracellular Recording

For extracellular recording, electrode internal solution was normal ACSF. The stimulating electrode was placed in layer 2/3, and the recording electrode was placed directly lateral in layer 5 of the prelimbic cortex, as visualized under 4× objective of a BX61WI microscope (Olympus America, Center Valley, PA, USA). Photomicrographs of electrode positions were taken and reviewed *post hoc* to ensure electrode placement remained consistent across the course of the experiment. In the presence of the NMDA receptor antagonist (2*R*)-amino-5-phosphonovaleric acid (APV; 50 μM) and the AMPA/kainate receptor antagonist 6-cyano-7-nitroquinoxaline-2,3-dione (CNQX; 6 μM) only the fiber volley persisted. In the presence of the voltage-gated sodium channel antagonist tetrodotoxin (TTX; 0.4 μM) both the presynaptic fiber volley and the field excitatory postsynaptic potential (fEPSP) were completely abolished. Both fiber volley and fEPSP measurements are negative-going peak amplitude values in relation to a pre-stimulus baseline level. (*n* = 20 slices from 12 sham-operated animals and *n* = 14 slices from 9 LFPI animals).

#### Whole-Cell Patch-Clamp Recording

In all whole-cell patch-clamp recordings, only one cell was recorded per brain slice. For whole-cell patch-clamp recording of spontaneous- and miniature excitatory postsynaptic currents (sEPSCs and mEPSCs) and intrinsic excitability measures electrode internal solution contained (in millimolar): K Gluconate 145, HEPES 10, BAPTA 0.1, NaCl 2.5, MgCl_2_ 2, Mg-ATP 2, GTP-Tris 0.5, and was titrated to a final pH of 7.2–7.3 with KOH. Bicuculline methiodide (30 μM) was added before voltage clamp recordings, and TTX (0.4 μM) was added to isolate mEPSCs. Liquid junction potential of 14.5 mV (calculated in Clampex) was corrected for in all data reported from these experiments. Neurons were voltage-clamped at −85 mV for all voltage clamp experiments of excitatory currents (Layer 2/3: *n* = 10 slices from 8 sham-operated animals and *n* = 9 slices from 8 LFPI animals. Layer 5: *n* = 22 slices from 13 sham-operated animals, and *n* = 23 slices from 13 LFPI animals). In order to reduce heterogeneity of neurons recorded, experiments began with a series of depolarizing current steps. If neurons fired non-accommodating trains of action potentials followed by large after-hyperpolarizations, they were deemed fast-spiking interneurons and excluded from further analysis. For whole-cell patch-clamp recording of inhibitory currents, internal solution contained (in millimolar): CsCl 130, MgCl_2_ 2, HEPES 10, BAPTA 0.1, Mg-ATP 2, GTP-Tris 0.5, and was titrated to final pH of 7.2–7.3 with CsOH. APV (50 μM) and CNQX (6 μM) were added before voltage clamp recordings, and TTX (0.4 μM) was added to isolate mIPSCs. Liquid junction potential of 4.1 mV (calculated in Clampex) was corrected for in all data reported from these experiments. Neurons were voltage-clamped at −74 mV for all voltage clamp experiments of inhibitory currents (Layer 2/3: *n* = 14 slices from 9 sham-operated animals and *n* = 18 slices from 10 LFPI animals. Layer 5: *n* = 14 slices from 9 sham-operated animals and *n* = 18 slices from 13 LFPI animals).

Patch electrodes with resistances of 4–6 MΩ were pulled from borosilicate glass (World Precision Instruments, Sarasota, FL, USA). Series resistance was monitored throughout the experiment and recordings were discontinued if series resistance exceeded 25 MΩ at any point. Series resistance was compensated for at 80% compensation. All recordings were made using a Multiclamp 700B (Molecular Devices, Palo Alto, CA, USA) sampled at 20 kHz, filtered at 2.4 kHz. Electrophysiological data were analyzed using Clampfit 10 (Molecular Devices) and MATLAB R2012b (Mathworks, Natick, MA, USA). Synaptic events were determined via the Template Search algorithm in Clampfit 10.

#### Intrinsic Excitability Measures

Resting membrane potential was computed as the average voltage in the first 2 s immediately after whole-cell configuration was achieved. All other intrinsic excitability measures were computed from current clamp recordings consisting of a series of ten 500 ms current steps, from −50 to 175 pA in 25 pA increments. Constant holding current was applied to maintain the neuron at −85 mV before/after current steps. Action potential threshold was computed by taking dV/dt of the voltage record in the intrinsic excitability experiments described in Figures 4 and 6. Threshold was defined as the point where dV/dt first exceeded 30 mV/ms (42). Input resistance was determined from the steady-state voltage response to the two hyperpolarizing steps (−50 and −25 pA) and the first depolarizing step (25 pA) (Layer 2/3: *n* = 17 slices from 9 sham-operated animals and *n* = 12 slices from 8 LFPI animals. Layer 5: *n* = 20 slices from 12 sham-operated animals and *n* = 14 slices from 9 LFPI animals).

### Immunohistochemistry

Seven days after LFPI or sham procedure, animals used in behavioral experiments were anesthetized with 5% chloral hydrate and perfused with 10 ml of saline, followed by 50 ml of paraformaldehyde (4% in phosphate buffer, pH 7.4; Sigma-Aldrich). Brains were post-fixed for 90 min at room temperature (RT) and 50 μm thick frontal (also known as coronal) sections were cut with a VT1000S vibratome (Leica Microsystems Inc.). For glial fibrillary acidic protein (GFAP) staining, sections were incubated with a rat monoclonal antibody (ascetic fluid) against GFAP (1:2 in phosphate-buffered saline [PBS]; gift from Dr. Judith Grinspan, CHOP) before visualization with Alexa Fluor 488–conjugated goat anti-rat IgG (1:200 in PBS; Molecular Probes). Primary antibody incubation was applied for 90 min at RT and continued overnight at 4°C, and the secondary antibody for 90 min at RT. Immunostained sections were counterstained with Hoechst. Confocal images were acquired with the Olympus Fluoview 1000 System (Olympus America), with the Z-step kept at 0.5 μm. Consistent confocal settings (laser intensity, confocal aperture, photomultiplier tube, gain, offset, and resolution) were optimized and remained unchanged during the imaging of slices from both sham and LFPI animals.

### Statistics

In analyzing the T-maze behavioral data, we controlled for individual variations in animal performance (i.e., the animal’s ability to perform the behavior before injury or sham procedure) by creating a measure we termed “Memory Performance.” We defined Memory Performance as the difference between the animal’s percent trials correct on the 3 days preceding LFPI or sham procedure and the percent trials correct on the post-injury day in question; thus, negative scores of Memory Performance are associated with a decrease in working memory ability. All behavioral data were confirmed to be Gaussian by D’Agostino-Pearson omnibus tests, and evaluated using Student’s *t*-tests.

Comparison of fEPSP data between LFPI mice and sham-operated controls was assessed in two ways. First, error ellipses demarcating the 95% confidence region of the group data at each stimulation intensity were generated with non-overlapping regions demonstrating significant differences. Additionally, we performed a non-parametric permutation-based bootstrapping analysis designed to correct for multiple comparisons. The bootstrapping analysis also allowed us to assess the fiber volley amplitude at which the fEPSP response was significantly different between LFPI mice and sham-operated controls. All individual data were initially pooled, and subjects were then randomly assigned to two groups with the same sizes as the initial comparison. Mean fiber volley and fEPSP amplitudes were computed at each stimulation intensity for each group. An exponential line of best fit was subsequently drawn through the mean data for each group. Next, the difference in fEPSP amplitude between the two fits was computed at intervals of.01 mV of fiber volley amplitude. This procedure was repeated 500 times, thus, creating a distribution of differences at each value of fiber volley amplitude, spaced by.01 mV. Finally, we performed the comparison using the actual data groups, took the group means, fit them with exponentials, computed the difference between the fits at the same intervals, and compared the differences to the distributions computed at each interval using a *Z*-test assessed at a *P* < 0.05 level. This analysis was performed in MATLAB R2012b (Mathworks).

All comparisons between LFPI mice and sham-operated controls from measures from whole-cell patch-clamp recordings were assessed by Mann–Whitney *U* tests, or repeated measures ANOVA where appropriate. Prism 6 (GraphPad Software, La Jolla, CA, USA) or SPSS 21 (IBM, Armonk, NY, USA) was used to perform these comparisons. In group data plots assessed by Mann–Whitney *U* tests, the median is presented. In group data plots assessed by repeated measures ANOVA, means and SEs of the mean are presented.

## Results

### LFPI Produces Working Memory Deficits in the T-maze

In order to assess the effect of brain injury on working memory, we performed a delayed non-match to sample task in the T-maze. By pre-training mice before injury, we were able to assess the ability to encode and retain a brief spatial memory trace, rather than the ability to learn the “rule” governing the behavioral paradigm. After training mice to perform the task, one subset of the mice received an LFPI, while the remaining mice received a sham procedure. We then tested the mice in this paradigm for the next seven consecutive days (for details, see Materials and Methods). In an effort to determine whether brain injury affected working memory following LFPI, we combined the data from days 1–7 post injury, and compared LFPI animals working memory performance to that of sham-operated controls. This revealed the decreased ability of brain-injured animals in this working memory task in the first week after brain injury (Sham 0.050 ± 0.017, LFPI −0.048 ± 0.023, *P* = 0.0008, Figure 1B). Next, we sought to determine whether the effects of injury were sustained and persisted into the second half of the post-injury testing period. In order to assess this, we binned the data into early (days 1–3 post-injury) and late (days 4–7 post-injury) periods after injury. We found that brain-injured mice showed a reduced ability in the working memory task at both early and late periods post injury (Early: Sham 0.030 ± 0.022, LFPI −0.067 ± 0.35, *P* = 0.018. Late: Sham 0.065 ± 0.026, LFPI −0.033 ± 0.030, *P* = 0.016, Figure 1C).

### LFPI Reduces Network Excitability in Prefrontal Cortex

**Figure 2:**
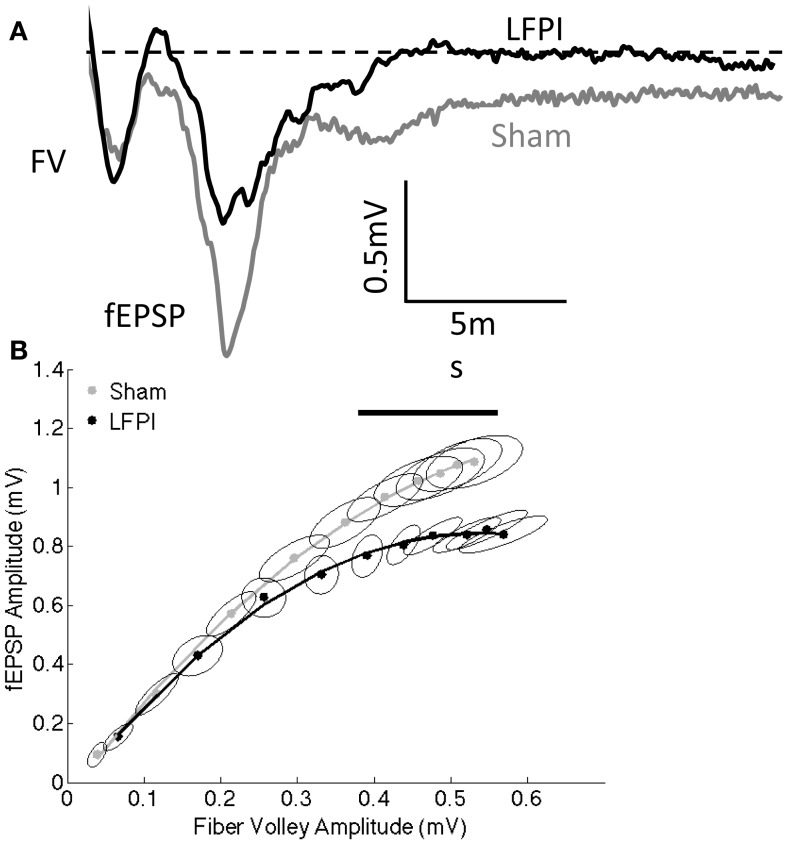
**Decreased field excitatory postsynaptic potentials following LFPI**. **(A)** Representative extracellular recordings at the highest stimulation intensity used (300 μA). Fiber volley amplitude is unchanged; however, fEPSP amplitude is reduced in response to LFPI. Dashed line indicates baseline voltage, aligned for both traces. FV denotes fiber volley, fEPSP denotes the field excitatory postsynaptic potential. **(B)** Fiber volley amplitude and fEPSP amplitude at stimulation intensities ranging from 30–300 μA. Error ellipses indicate the 95% confidence area for each measure. The horizontal line above the data indicates the fiber volley amplitudes for which the groups differ in fEPSP amplitude as assessed by a permutation-based bootstrapping method at the α = 0.05 level.

Proper functioning of neuronal circuits requires a balance between synaptic excitation and inhibition (E/I balance). In order to assess potential changes in E/I balance on a network level, we performed extracellular recordings in mPFC an area that has been shown to be important for working memory in the T-maze non-match to sample paradigm as well as similar behavioral tasks (25, 43, 44). We stimulated layer 2/3 of the mPFC and measured the extracellular response in mPFC layer 5. The signal that is produced has two components: the fiber volley, reflecting presynaptic action potentials, and the fEPSP, which results from voltage changes in the postsynaptic dendrites (Figure 2A). We stimulated over a range of intensities, thereby producing an input–output curve. We observed a reduction in the fEPSP after LFPI, without a significant change in the size of the fiber volley (Figure 2B), indicating that the decrease in the fEPSP was not due to a reduction in the number of afferent fibers stimulated. Data were analyzed both through the generation of 95% confidence interval (CI) error ellipses and a permutation-based bootstrapping analysis in order to correct for multiple comparisons (for details, see Materials and Methods). Both analyses revealed a decrease in fEPSP amplitude in slices derived from LFPI animals, demonstrating a significant decrease in network excitability in the mPFC in response to LFPI.

### LFPI-Induced Changes in Synaptic Transmission in Layer 2/3 of Prefrontal Cortex

In order to investigate the source of the LFPI-induced decrease in network excitability described above, we performed a series of whole-cell patch-clamp recordings to measure spontaneous and miniature postsynaptic currents. Miniature currents were isolated by addition of tetrodotoxin to the extracellular solution, thereby eliminating action potentials. Increases or decreases in miniature excitatory currents in the mPFC have been shown to play a role in behavior (31) and we hypothesized that these action potential-independent currents may contribute to the observed changes in working memory and network excitability.

**Figure 3:**
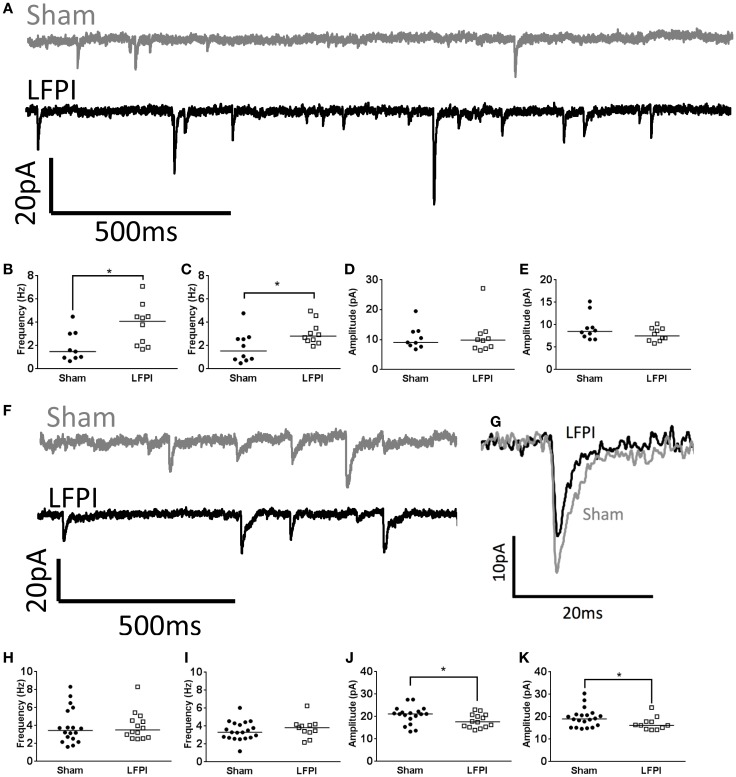
**Shifts in synaptic input onto layer 2/3 following LFPI**. **(A)** Representative traces from layer 2/3 mPFC neurons showing sEPSCs, note the increase in frequency of currents in the LFPI trace. **(B)** Group data illustrating increase in frequency of sEPSCs and **(C)** mEPSCs in LFPI neurons (* indicates *P* < 0.05). **(D)** Group data illustrating no difference in amplitude of sEPSCs or **(E)** mEPSCs following LFPI. **(F)** Representative traces from layer 2/3 mPFC neurons showing sIPSCs. **(G)** Representative traces of a single sIPSC of median amplitude. Note the decrease in amplitude after LFPI. **(H)** Group data illustrating no change in frequency of sIPSCs or **(I)** mIPSCs in LFPI neurons. **(J)** Group data illustrating increase in amplitude of sIPSCs and **(K)** mIPSCs following LFPI (* indicates *P* < 0.05).

In brain slices derived from LFPI mice, the frequency of sEPSCs onto layer 2/3 neurons was increased (95% CI Sham 0.913–2.93 Hz, LFPI 2.48–5.05 Hz, *P* = 0.0133, Figures 3A,B). The frequency of mEPSCs also increased in slices derived from LFPI animals (95% CI Sham 0.897–2.81 Hz, LFPI 2.36–3.79 Hz, *P* = 0.0431, Figure 3C). There was no change in the amplitudes of sEPSCs (95% CI Sham 7.675–13.72 pA, LFPI 6.698–15.37 pA, *P* = 0.7655, Figure 3D), or mEPSCs (95% CI Sham 7.294–11.42 pA, LFPI 6.758–8.837 pA, *P* = 0.1883, Figure 3E). Similarly, the charge transfer of the currents, as assessed by the area under the curve, was unchanged by injury (sEPSCs; 95% CI Sham 32.70–52.51 pA*ms, LFPI 32.19–55.89 pA*ms, *P* > 0.9999. mEPSCs; 95% CI Sham 33.30–44.24 pA*ms, LFPI 29.73–43.76 pA*ms, *P* = 0.6706, data not shown). Conversely, the frequency of spontaneous inhibitory postsynaptic currents (sIPSCs, 95% CI Sham 2.98–4.94 Hz, LFPI 3.03–4.86 Hz, *P* = 0.7724) and miniature inhibitory postsynaptic currents (mIPSCs, 95% CI Sham 2.96–3.94 Hz, LFPI 3.05–4.48 Hz, *P* = 0.5528) were unaffected by LFPI (Figures 3F,H,I). However, the amplitude of sIPSCs (95% CI Sham 18.68–22.62 pA, LFPI 16.35–19.85 pA, *P* = 0.0489) and mIPSCs (95% CI Sham 17.61–21.51 pA, LFPI 14.96–18.96 pA, *P* = 0.0350) were decreased in slices derived from LFPI mice (Figures 3G,J,K). The charge transfer of sIPSCs was decreased in LFPI mice (95% CI Sham 182.9–234.0 pA*ms, LFPI 132.9–178.1 pA*ms, *P* = 0.0078, data not shown). However, the charge transfer of mIPSCs was not significantly different in LFPI mice (95% CI Sham 174.2–215.4 pA*ms, LFPI 150.9–196.2 pA*ms, *P* = 0.1306, data not shown).

### Intrinsic Excitability of Layer 2/3 Neurons is Affected by LFPI

**Figure 4:**
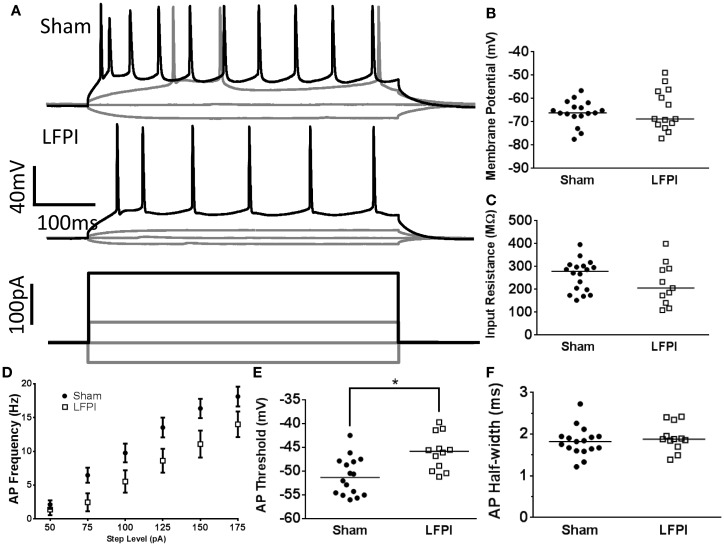
**Alterations in layer 2/3 intrinsic excitability following LFPI**. **(A)** Representative traces from layer 2/3 neurons showing voltage response to selected current steps (−50, 0, 50, 175 pA). Response to 175 pA current step shown in black for visual clarity. Note the decreased firing rate in the LFPI trace. **(B)** Group data displaying no change in resting membrane potential after LFPI. **(C)** Group data illustrating no difference in input resistance after LFPI. **(D)** Frequency versus current group data showing an increase in firing rate following LFPI. **(E)** Action potential threshold is significantly depolarized in slices derived from LFPI animals (* indicates *P* < 0.05). **(F)** Action potential half-width is unchanged following LFPI.

Neuronal excitability is influenced by both afferent synaptic excitation/inhibition balance as well as the intrinsic excitability of the neuron itself, which is chiefly determined by the neuron’s own cohort of membrane proteins and their activity. In order to assess the intrinsic excitability of layer 2/3 neurons, we subjected each neuron to a series of hyperpolarizing and depolarizing current steps (10 steps, 25, −50 to +175 pA, 500 ms, Figure 4A). Resting membrane potential was unchanged in neurons recorded from LFPI slices (95% CI Sham −68.7 to 63.4 mV, LFPI −70.1 to 59.9 mV, *P* = 0.9093, Figure 4B). Input resistance was similarly unaffected by LFPI (95% CI Sham 226.2–294.3 MΩ, LFPI 161.9–285.4 MΩ, *P* = 0.2328, Figure 4C). However, neurons in slices derived from LFPI animals responded with significantly fewer action potentials in response to varying levels of current injection [*F*(1,28) = 4.469, *P* = 0.044, Figure 4D] and the action potential threshold was elevated in slices derived from LFPI animals (95% CI Sham −53.2 to 48.9 mV, LFPI −48.4 to 43.6 mV, *P* = 0.0022, Figure 4E). However, action potential duration, as determined by the half-width, was unaffected by LFPI (95% CI Sham 1.645–2.002 ms, LFPI 1.713–2.131 ms, *P* = 0.3674, Figure 4F) as was action potential height (95% CI Sham 106.7–117.1 mV, LFPI 104.2–120.2 mV, *P* = 0.9761, data not shown).

### LFPI Does Not Affect Spontaneous Synaptic Transmission in Layer 5 of Prefrontal Cortex

In order to test whether the output of layer 2/3 neurons was maintained after LFPI, we recorded spontaneous and miniature excitatory currents in layer 5 neurons. The intra-cortical projection from layer 2/3 to layer 5 is well established, by its ability to generate fEPSPs resembling those evoked in Schaffer collaterals and recorded in stratum radiatum of CA1 (45), classical labeling studies using retrograde tracers (46) and paired recording (47, 48). We hypothesized that there would not be any change in sEPSCs in layer 5 after LFPI, despite the increase in excitatory activity onto layer 2/3 neurons due to the elevation of the action potential threshold in the layer 2/3 neurons following LFPI.

**Figure 5:**
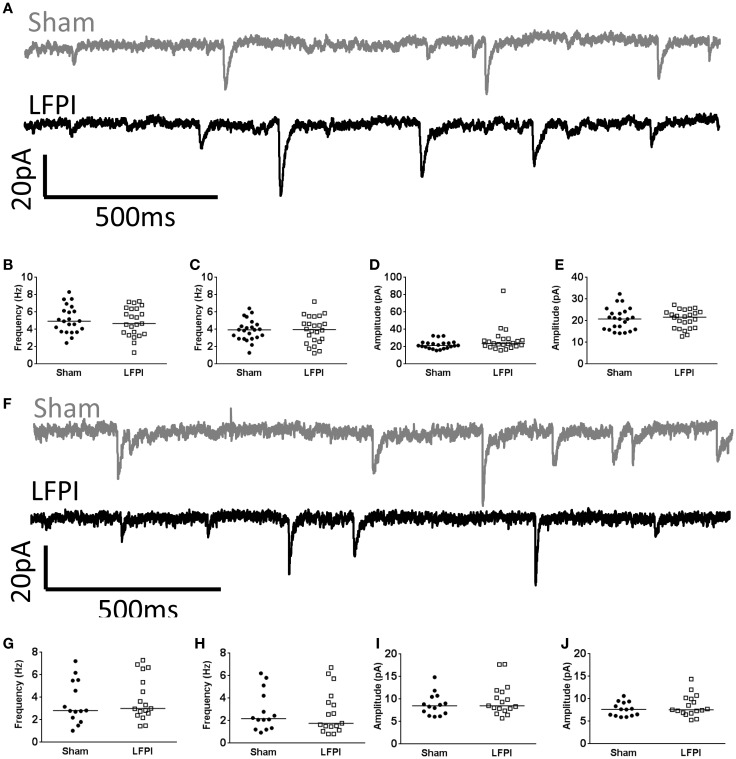
**Synaptic input to layer 5 is unchanged following LFPI**. **(A)** Representative traces from layer 5 mPFC neurons showing sEPSCs. **(B)** Group data illustrating no change in frequency of sEPSCs or **(C)** mEPSCs in LFPI neurons. **(D)** Group data illustrating no difference in amplitude of sEPSCs or **(E)** mEPSCs following LFPI. **(F)** Representative traces from layer 5 mPFC neurons showing sIPSCs. **(G)** Group data illustrating no change in frequency of sIPSCs or **(H)** mIPSCs in LFPI neurons. **(I)** Group data illustrating no difference in amplitude of sIPSCs or **(J)** mIPSCs following LFPI.

In contrast to layer 2/3, and in support of the prediction, all measured parameters of sEPSCs remained unchanged in brain slices derived from LFPI mice (Figure 5A, Frequency: 95% CI Sham 2.45–4.65 Hz, LFPI 2.86–4.87 Hz, *P* = 0.6154. Amplitude: 95% CI Sham 7.385–10.26 pA, LFPI 8.074–11.69 pA, *P* = 0.4855. Area: 95% CI Sham 35.73–60.36 pA*ms, LFPI 40.58–52.95 pA*ms, *P* = 0.6712, Figures 5B,D). Similarly, parameters of mEPSCs were unaffected by LFPI (Frequency: 95% CI Sham 1.82–3.86 Hz, LFPI 1.76–3.74 Hz, *P* = 0.7036. Amplitude: 95% CI Sham 6.790–8.567 pA, LFPI 7.202–9.666 pA, *P* = 0.4614. Area: 95% CI Sham 37.16–44.07 pA*ms, LFPI 36.60–46.73 pA*ms, *P* = 0.7581, Figures 5C,E). We also measured inhibitory currents in layer 5 neurons, as we hypothesized there may be a change in feed-forward inhibition onto layer 5 neurons. However, all measured parameters of sIPSCs (Figure 5F, Frequency: 95% CI Sham 4.43–5.83 Hz, LFPI 4.15–5.59 Hz, *P* = 0.6564. Amplitude: 95% CI Sham 20.20–24.53 pA, LFPI 21.36–33.47 pA, *P* = 0.1311. Area: 95% CI Sham 207.7–266.0 pA*ms, LFPI 220.0–351.1 pA*ms, *P* = 0.2083, Figures 5G,I) and mIPSCs (Frequency: 95% CI Sham 3.36–4.43 Hz, LFPI 3.26–4.58 Hz, *P* = 0.9183. Amplitude: 95% CI Sham 18.56–23.07 pA, LFPI 18.98–22.50 pA, *P* = 0.8029. Area: 95% CI Sham 182.3–228.8 pA*ms, LFPI 192.8–230.1 pA*ms, *P* = 0.8685, Figures 5H,J) remained unchanged as well.

### Intrinsic Properties of Layer 5 Neurons Following LFPI

While the synaptic currents onto layer 5 neurons were not changed following LFPI, membrane proteins in layer 5 neurons may change after LFPI, thus, altering cellular intrinsic excitability. In order to examine this possibility, we measured intrinsic excitability by injecting the same series of current steps as in the layer 2/3 experiments described in Figure 4 (10 steps, 25, −50 to +175 pA, 500 ms).

**Figure 6:**
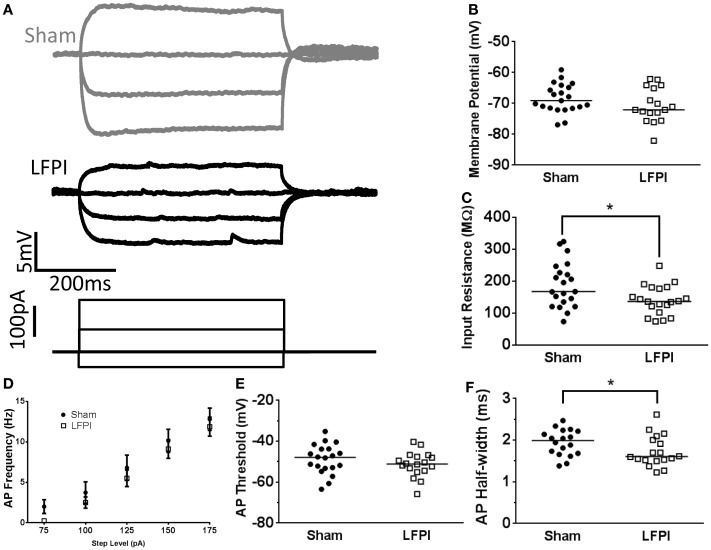
**Input resistance of layer 5 neurons is affected by LFPI**. **(A)** Representative traces from layer 5 neurons showing voltage response to selected current steps (−50, −25, 0, 25 pA). **(B)** Group data displaying no change in resting membrane potential after LFPI. **(C)** Group data showing a reduction in input resistance in layer 5 neurons after LFPI (* indicates *P* < 0.05). **(D)** Frequency versus current group data shows no change in firing rate after LFPI. **(E)** Action potential threshold is unchanged after LFPI. **(F)** Group data, illustrating significantly shorter action potential half-width following LFPI (* indicates *P* < 0.05).

First, we measured resting membrane potential and found that it was unaffected by LFPI (95% CI Sham −70.5 to 66.3 mV, LFPI −73.5 to 67.8 mV, *P* = 0.1669, Figure 6B). We did, however, note a decrease in input resistance of layer 5 neurons after LFPI (95% CI Sham 156.3–220.8 MΩ, LFPI 119.6–162.2 MΩ, *P* = 0.0369, Figure 6C). Generically, a decrease in input resistance results from increased membrane permeability, though it is unclear what the molecular mechanism for this increased permeability may be. We also observed a decrease in the duration of the action potential after LFPI, as measured by action potential half-width (95% CI Sham 1.789–2.099 ms, LFPI 1.550–1.916 ms, *P* = 0.0468, Figure 6F). A decrease in input resistance often results in a decrease in intrinsic excitability; however, this was not the case in the layer 5 neurons we recorded. Specifically, the change in input resistance was not accompanied by a change in firing rate [*F*(1,33) = 0.454, *P* = 0.505, Figure 6D] or action potential threshold (95% CI Sham –52.2 to 45.5 mV, LFPI −54.4 to 48.2 mV, *P* = 0.2798, Figure 6E). Action potential height was also unaffected by LFPI (95% CI Sham 103.1–115.3 mV, LFPI 103.9–112.3 mV, *P* = 0.6213, data not shown).

### Reactive Astrocytosis in Ipsilateral but Not Contralateral mPFC After LFPI

**Figure 7:**
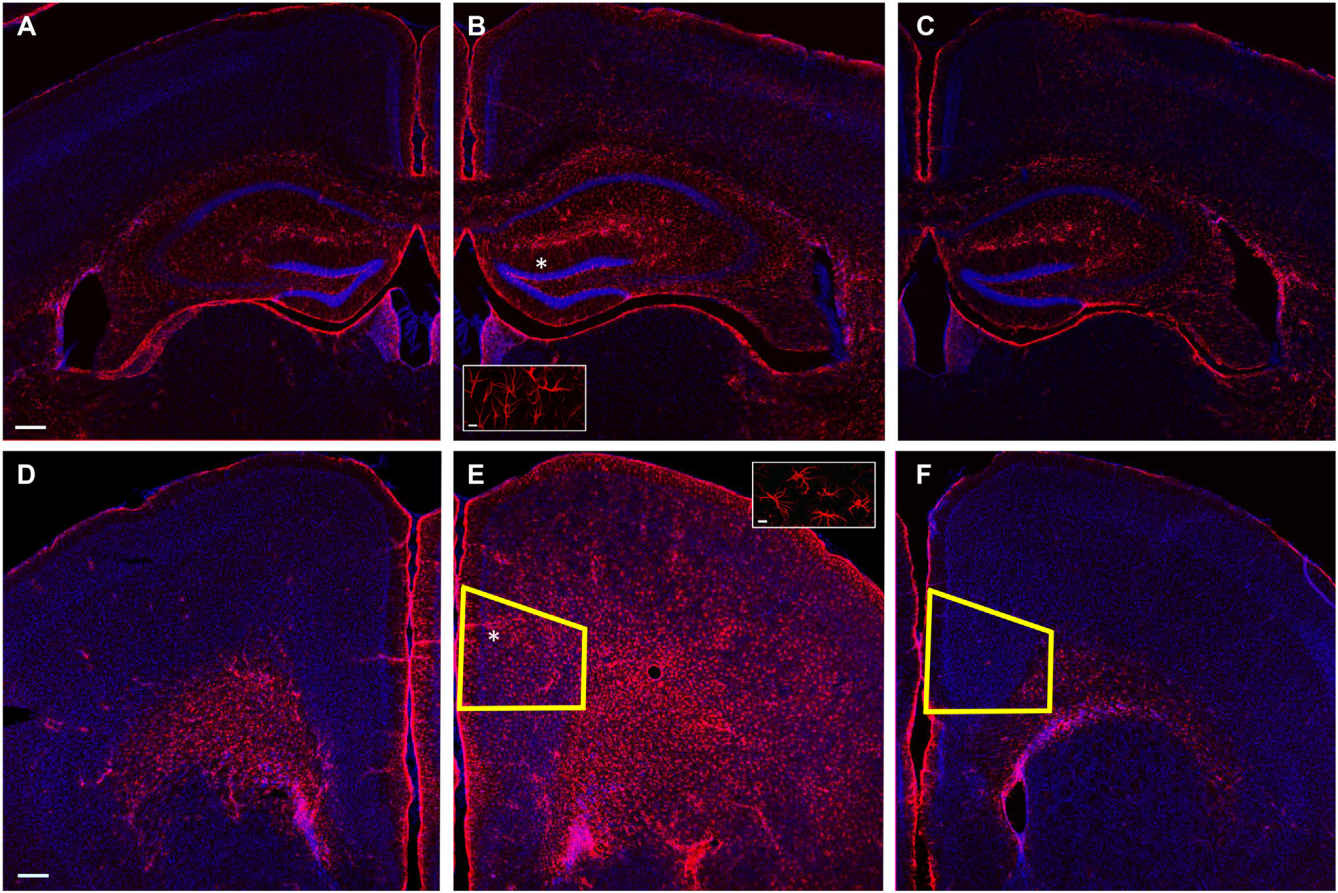
**GFAP labeling following LFPI**. **(A)** Example photomicrograph of contralateral hippocampus from LFPI animal. **(B)** Example photomicrograph of hippocampus ipsilateral to LFPI, inset displaying astrocyte morphology. **(C)** Example photomicrograph of ipsilateral hippocampus from sham-operated animal. **(D)** Example photomicrograph of contralateral mPFC from LFPI animal. **(E)** Example photomicrograph of mPFC ipsilateral to LFPI, inset displaying astrocyte morphology. **(F)** Example photomicrograph of ipsilateral mPFC from sham-operated animal. GFAP labeling in red, Hoescht labeling for DNA in blue. Scale bars: 200 μm in **(A)**–**(F)**; 10 μm in insets. Asterisk (*) indicates region selected for inset. Yellow trapezoid in **(E,F)** outlines prelimbic cortex, where all physiology experiments were performed.

Lateral fluid percussion injury produces both focal and diffuse effects, with brain regions immediately under the injury site experiencing the most damage, while more distant parts of the brain, including the contralateral hemisphere, are relatively spared (12, 49, 50). We predicted that the mPFC is far enough removed from the LFPI site to escape immediate damage from the pressure wave that causes the injury and any circuit deficits observed could be attributed to secondary effects, as is the case in the contralateral hippocampus (50). In particular, we reasoned that if the mPFC was indeed too distant to be directly affected by LFPI, it would not show signs of reactive astrocytosis, which has been shown consistently in the ispilateral hippocampus, but not the contralateral hippocampus following mild to moderate experimental brain injury (51, 52). We stained slices from LFPI animals and sham-operated controls with an antibody to GFAP, a commonly used marker of reactive astrocytosis. Consistent with previous reports employing mild to moderate experimental brain injury, we observed intense GFAP staining in the ipsilateral hippocampus of LFPI animals (Figure 7B), while contralateral hippocampus displayed modest GFAP staining (Figure 7A). Similarly, modest staining to that observed in the contralateral hippocampus was observed in the ipsilateral hippocampus in sham-operated control animals (Figure 7C). In order to examine whether the mPFC experiences a similar increase in reactive astrocytosis after LFPI, we performed similar staining experiments in mPFC. In contrast to our hypothesis, we found intense GFAP staining in the ipsilateral mPFC of LFPI animals (Figure 7E) and sparse staining in the mPFC contralateral to the LFPI (Figure 7D). In slices from sham-operated controls, we observed little or no GFAP staining (Figure 7F). This pattern of ipsilateral reactive astrocytosis supports the notion that the effects of injury in the ipsilateral mPFC are similar to that of the ipsilateral hippocampus.

## Discussion

In this study, we demonstrate working memory impairments acutely following mild to moderate LFPI and sustained for 7 days post-injury. Initially, we show a reduction in network excitability in the mPFC, a brain region involved in working memory, 7 days post-injury. Additionally, further experiments reveal shifts in both excitatory and inhibitory synaptic transmission that are specific to layer 2/3. Furthermore, we present changes in the intrinsic excitability of cells in both layer 2/3 and layer 5 of the mPFC.

Previous reports employing LFPI to describe working memory impairment in rats have focused on later time points (beginning at ~14 days) and have shown a delay-dependent deficit, unlike the current study (14, 16). The experiments presented here were designed to investigate physiological changes within the clinically relevant “therapeutic time window” but after transient changes that subside by 48 h post-injury (53). Working memory deficits are a constant finding across multiple models of TBI (15, 22, 54), and here we illustrate an acute and enduring impairment of working memory using a common and well-characterized experimental model of TBI and species/strain of rodent.

To probe the underlying substrate contributing to the behavioral impairment, we performed experiments that illustrate a series of physiological alterations in mPFC circuitry 7 days after LFPI. Our initial physiological finding, a decrease in network excitability in the mPFC, as shown by the decrease in fEPSPs, demonstrates a shift in E/I balance in this circuit following LFPI. Shifts in E/I balance have been previously reported following LFPI in the hippocampus, and reinstatement of this balance has been effective in restoring hippocampal-dependent cognition (19). This report is the first to describe E/I imbalances in the mPFC following LFPI.

In order to examine potential circuit mechanisms that could be contributing to shifts in network excitability after brain injury, we measured both excitatory and inhibitory currents onto layer 2/3 neurons as well as the intrinsic membrane properties of layer 2/3 neurons. These experiments demonstrated a net increase in the excitatory drive onto layer 2/3 neurons, potentially making these neurons more likely to fire action potentials. However, examination of intrinsic excitability parameters determined that action potential threshold was increased. This produced the opposite effect as layer 2/3 neurons fired less action potentials in response to current injection. This type of compensatory response has been well described in other paradigms (55, 56) as well as in other brain regions after LFPI (42). Unlike the previous work investigating homeostasis after brain injury, which also described opposing shifts in intrinsic excitability and synaptic inputs after LFPI, this study employs behavioral testing to assess the behavioral relevance of these changes. The behavioral data supports the notion that the potential compensatory mechanisms we observed were insufficient in mitigating cognitive impairment; however, more experiments will be needed to support this hypothesis. Additionally, further experiments are necessary to verify that post-LFPI alterations in intrinsic excitability are a compensatory response to changes in synaptic input and not a direct effect of the injury itself.

There are multiple biological mechanisms that may contribute to differential vulnerability of layer 2/3 and layer 5 neurons in the mPFC after brain injury. Chiefly, there are two distinct subtypes of excitatory output neurons within the mPFC, pyramidal tract (PT), and intratelencephalic (IT) neurons, which are defined by their projection targets (57–59). IT neurons are found in both layer 2/3 and layer 5, while PT neurons are only found in layer 5. Importantly, these two classes of neurons respond differentially to neuromodulators, including dopamine and serotonin (60, 61). Furthermore, IT neurons and PT neurons express different cohorts of intrinsic membrane channels, which affect the way the two classes of neurons respond to network input and lead to each type having a unique role within the prefrontal circuit (48, 62). Modifying these intrinsic properties, specifically h-current, has been shown to cause both improvements and deficits in memory performance (63, 64). The current study supports a possible preferential alteration of IT neurons to injury. However, the molecular mechanism(s) underlying this sensitivity and the observed insensitivity of PT neurons is a topic for further exploration.

To our knowledge, this is the first study describing working memory deficits acutely and continuously throughout the first week after LFPI. The data demonstrate no evidence of recovery of working memory in the 7 days following brain injury. Unlike previous studies that have focused on morphological and neurochemical changes, this study assesses physiological correlates of working memory dysfunction in the prefrontal cortex. These results support the hypothesis that injury-induced disruption of E/I balance is a key factor contributing to behavioral impairments. Additionally, our results support the idea that the prefrontal cortex, along with other regions known to be affected by brain injury, plays a role in post-injury memory deficits. Future studies could extend the current investigation into the cellular and molecular mechanisms of the observed changes as well as further bridging the gap between physiology and behavior by recording from the prefrontal cortex in awake-behaving animals after LFPI.

## Author Contributions

CS designed experiments; collected, analyzed, and interpreted data; and wrote and revised the manuscript. GX, JE, and BP collected data and revised the manuscript. AC designed experiments, edited, and revised the manuscript. All authors approved the final version and are accountable for all aspects of the work.

## Conflict of Interest Statement

The authors declare that the research was conducted in the absence of any commercial or financial relationships that could be construed as a potential conflict of interest.
